# Effect of heavy load carriage on cardiorespiratory responses with varying gradients and modes of carriage

**DOI:** 10.1186/s40779-018-0171-8

**Published:** 2018-07-26

**Authors:** Subhojit Chatterjee, Tirthankar Chatterjee, Debojyoti Bhattacharyya, Suranjana Sen, Madhusudan Pal

**Affiliations:** Defence Institute of Physiology and Allied Sciences, Defence Research & Development Organisation, Ministry of Defence, Government of India, Lucknow Road, Timarpur, Delhi, 110054 India

## Abstract

**Background:**

The present study was undertaken to determine the effect of different uphill and downhill gradients on cardiorespiratory and metabolic responses of soldiers while carrying heavy military loads in two different modes.

**Methods:**

Eight physically fit male soldiers with a mean age 32.0 ± 2.0 years, a mean height of 169.5 ± 4.9 cm, and a mean weight of 63.8 ± 8.4 kg volunteered for this study. Each volunteer completed treadmill walking trials at a speed of 3.5 km/h while carrying no external load, 31.4 kg load in a distributed mode (existing load carriage ensembles) and compact mode (new back pack) over 5 different downhill and uphill gradients (− 5, − 10%, 0, 5, 10%) for 6 min at each gradient. During the walking trials, heart rate (HR), oxygen uptake (VO_2_), respiratory frequency (RF) and energy expenditure (EE) were determined by the process of breath-by-breath gas analysis using a K4b^2^ system. The average of the last 2 min data from each 6 min walking trial for each individual was subjected to statistical analysis.

**Results:**

All parameters (HR, VO_2_, RF, and EE) gradually increased with the change in gradient from downhill to level to uphill. The distributed mode showed higher values compared to compact mode for all gradients, e.g., for VO_2_, there was a 10.7, 7.4, 5.1, 28.2 and 18.7% increase in the distributed mode across the 5 different gradients.

**Conclusion:**

It can be concluded from the present study that the compact mode of load carriage is more beneficial than the distributed mode in terms of cardiorespiratory responses while walking on downhill and uphill surfaces with a 31.4 kg load.

## Background

Heavy load carriage is a common phenomenon in military operations; Indian soldiers have to carry heavy loads over different terrains and in extreme environmental conditions. The distributed mode of load carriage (existing load carriage ensembles, ELCes) can support a load of up to 21.4 kg. These ELCes are composed of three components: a backpack (BP, 10.7 kg), which is the main unit and contains regular items such as the uniform, PT shoes, rations; the haversack (HS, 4.4 kg), which is the small unit for carrying essential items such as dry foods, medicine, and first aid; and the web (2.1 kg), which consists of the magazine, and along with this rifle (4.2 kg) is carriaed in hand. Irrespective of the occupational necessity for different operations, the load distribution generally allows the BP to be placed at the back, the HS is placed at the waist region and tied with the belt at the bottom of the BP or on either side of the body, and the web is located on the front of the body. This is an unequal load distribution, which can cause discomfort in the body of the carriers. Different researchers have found that the front pack - backpack combination is most economical in terms of energy cost. However, the weight distribution in this front pack (web) and BP (main BP) combination is also unequal in the ELCes. This type of distributed mode (DM) of load carriage creates more problems with the external frame, as the heavy weight of the BP rests on the frame which presses hard on the skin and muscle underneath. Consequently the soldiers feel discomfort, which in long run results in blisters, soreness and even in some critical musculoskeletal disorders. Additionally, carrying the rifle by hand in this manner restricts the arm’s natural swinging movement. Research has shown that restricted arm movement causes deviation of body’s center of mass (COM) from its normal path, which leads to higher energy expenditure [1–4].

Different modes of load carriage have been thoroughly investigated by several researchers in the past [4–14]. Considerable research has been carried out to determine the best method of load carriage that minimizes physical stress on the body [1, 8, 15–19]. However, studies regarding the physiological effects of load distribution on different parts of the body are less in number [20–25]. In earlier studies conducted by Pal et al. [10] and Chatterjee et al. [1], the load was placed as a single unit, such as a compact backpack. They compared the effect of carrying a 21.4 kg load in compact mode (CM) and distributed mode and found that the energy cost was higher in distributed mode when walking at a speed of 4.5 km/h at 5 and 10% gradients. The effect of downhill (DH) walking in combination with heavy load carriage using different modes had been studied less in the field of load carriage research.

In a walking trial carried out in the early 1970s, a greater VO_2_ drift over time was observed during DH gradients compared to level walking. The authors argued that while walking on a DH gradient the supporting muscles performed eccentric contractions, which caused recruitment of additional muscle fibers to maintain stability, that might have led to an increased VO_2_ [25]. The physiological study of sloped walking by Margaria et al. [26] found that during UH walking at a constant speed, the metabolic rate increased linearly, but during DH walking, the metabolic rate decreased until approximately − 6% gradients and then became higher for − 9% gradients and steeper slopes. More recently, in a study conducted by Santee et al., a reduction in VO_2_ during short duration load carriage was observed while walking on a DH gradient compared to level (0%) walking [27]. The lowest VO_2_ was reported  at − 8%, whereas further elevation in DH gradient caused an increase in VO_2_ [27]. Similar observations were made by Blacker et al. [28] and Chatterjee et al. [29]. However, only a few number of studies have compared different modes of load carriage (single compartmental load and loads distributed on various parts of the body) in combination with variations in slope ranging from DH walking (up to − 10% gradient) to UH walking (up to 10% gradient). Hence, a comparison of the effect of the two different modes of load carriage during UH (positive work) as well as DH (negative work) will be of relevance for the soldiers as they are the ones' who are regularly exposed to such working conditions. Thus, a study covering these factors (modes and slope) seems necessary for better understanding of the relationship of terrain complexity and proper distribution of load during carriage.

This study was designed to measure the effect of heavy load carriage in different DH and UH walking conditions and the effect of two different modes of carriage (compact and distributed mode) on Indian soldiers in terms of cardio-respiratory responses.

## Methods

### Participants

Eight physically fit male soldiers (with mean ± SD age of 32.0 ± 2.0 years, height of 169.5 ± 4.9 cm, and weight of 63.8 ± 8.4 kg) with at least 4 years of military service experience volunteered for this study. The participants had no history of musculoskeletal or cardiovascular pathology.

### Ethical clearance

An ethical clearance was obtained in prescribed format from the Ethical Clearance Committee of Defence Institute of Physiology and Allied Sciences, Defence Research and Development Organisation, in accordance with the Declaration of Helsinki (Helsinki, 1993). All volunteers were briefed about the purpose, risks and benefits of this study. An informed consent was obtained after this briefing.

### Experimental protocol

On the day of the load carriage experiment, the participants reported to the laboratory at 8:30 am after a light breakfast. The participants were instructed to wear their combat uniform along with their helmet and military boots. Participants were allowed to take 1 h of rest before the start of the experiment.

A controlled laboratory environment was created by maintaining the temperature at 22 °C to 27 °C and relative humidity at 45–55%. These environmental parameters were monitored through a digital thermometer-hygrometer. A motorized treadmill (model: M/S/Mercury 4,h/p/cosmos, GmBH, Leipzig, Germany) was used for the simulated walking task. Load carriage experiments were carried out on each participant by allowing them to carry a 31.4 kg load in the DM and CM while walking on a treadmill at a speed of 3.5 km/h for five different gradients that consisted of both DH and UH walking. The selection of speed of (3.5 km/h) was based on previous research experiences from the same lab and the findings of other researchers conducting similar load carriage tasks [30–32]. On the other hand, a walking speed of 3.5 km/h, on 10% gradient with a load of 31.4 kg represents strenuous physical work, if continued for a longer duration. The Indian army soldiers typically carry a load of approximately 21.4 kg as the standard for all terrains. The extra 10 kg load was applied in this study as the soldiers are often required to carry extra ammunition, rations, and bullet proof jackets based on emergency situations and long duration patrolling duties. This load is also close to 50% of the soldier’s body mass. The Armies of other developed nations, e.g., the soldiers in the US Army, carry a fighting load of 48 pounds (21.7 kg) and an approach march load of 72 pounds (32.6 kg), which is similar to the load applied in the present study [33].

Experiment started with the − 10% DH gradient, followed by − 5%, level walking at 0%, and then UH walking on slopes of 5 and 10% gradients. Each participant carried out this task continuously for 30 min, which consisted of walking at each gradient for 6 min. The duration of load carriage at each gradient was fixed at 6 min as any physically fit individual takes approximately 4 min to reach steady state while performing the same exercise at the same intensity [10]. This study design is supported by previous research in similar areas carried out in the same laboratory [29, 34].

The rifle was carried in hand for distributed mode, and it was kept in a rifle carrying pocket with attachments in the compact mode. Participants walked at the same speed for all gradients and completed a trial without load (no load, NL) at a similar duration as a control condition. The rotation of the treadmill belt had to be switched to the opposite direction for the UH gradients after completion of walking at the − 10, − 5% and 0% gradients. The participants were required to walk on another treadmill at 0% during the time taken for switching of the belt rotation.

A total of 88 experiments (5 gradients × 2 modes of carriage × 8 participants) + (8 NL condition) were carried out. Each participant was required to complete the two conditions within one week (between 08:30 am to 01:00 pm).

### Recording of cardiorespiratory parameters

Heart rate (HR, beats/min), oxygen uptake [VO_2,_ ml/(min·kg)], energy expenditure (EE, kcal/min) and respiratory frequency (RF, breaths/min) of all of the participants were determined by the process of breath-by-breath gas analysis using a K4b^2^ system (K4b^2^, Cosmed, S.R.L, Italy) throughout the experiments. For all of the above mentioned parameters the average value of last 2 min for each of the 6 min at each gradient were used for data processing and subjected to statistical analysis [31, 34].

### Statistical analysis

Repeated measures ANOVA was applied to determine the overall significance across the conditions for all the parameters, as all participants were exposed to all 5 gradients and 3 different modes of load carriage. Bonferroni’s post hoc test was applied for pairwise comparisons between the conditions. For all the tests, statistical significance was verified at *P* < 0.05. The statistical analysis was performed using SPSS software V.20 (IBM, USA).

## Results

It was observed that HR (beats/min), VO_2_ [ml/(min·kg)], RF (breaths/min), and EE (kcal/d) gradually increased from DH to level walking and from level walking to walking on the UH gradients. It was observed that during carriage of the 31.4 kg load by the DM and CM, cardio-respiratory parameters significantly increased compared to the no load condition when walking on all the measured gradients. The cardio-respiratory responses were lower with the CM as compared to the DM for all the gradients. Changes in HR, VO_2_, RF, and EE along with % changes across the conditions are presented in Table 1. The overall significance along with the significance level after pairwise comparisons across the conditions is presented in Table 2. The results of the ANOVA are explained with following abbreviations: G_1_- Grade 1, − 10%; G_2_- Grade 2–5%; G_3_- Grade 3, 0%; G_4_- Grade 4, 5%; G_5_- Grade 5, 10%; M_1_- Mode 1, NL; M_2_- Mode 2, distributed mode; and M_3_- Mode 3, compact mode.

**Table 1:** Physiological responses during load carriage with the DM and CM of the backpack

Parameter	Grade	Mode of carriage (mean ± SD)			% of change		
		No Load	DM (31.4 kg)	CM (31.4 kg)	NL vs DM	NL vs CM	CM vs DM
HR (beats/min)	−10%	88.00 ± 8.89	107.00 ± 7.00	92.00 ± 5.43	21.59^*^	4.55	16.30
	−5%	89.00 ± 8.01	111.00 ± 5.54	92.00 ± 4.91	24.72^*^	3.37	20.65^*^
	0%	95.00 ± 7.00	121.00 ± 4.67	100.00 ± 5.54	27.37^*^	5.26^*^	21.00
	5%	111.00 ± 8.31	160.00 ± 13.50	125.00 ± 8.73	44.14^*^	12.61^*^	28.00^*^
	10%	130.00 ± 9.09	183.00 ± 7.48	156.00 ± 8075	40.77^*^	20.00^*^	17.31
VO_2_ [ml/(min·kg)]	−10%	6.64 ± 2.17	9.12.00 ± 3.35	8.24 ± 2.05	37.35^*^	24.10	10.68
	−5%	6.76 ± 1.87	9.58 ± 3.85	8.92 ± 2.14	41.72^*^	31.95	7.40
	0%	9.45 ± 2.13	13.50 ± 3.39	12.84 ± 2.50	42.86^*^	35.87	5.14
	5%	15.14 ± 2.80	22.76 ± 3.57	17.75 ± 1.43	50.33^*^	17.24	28.23
	10%	21.12 ± 2.58	30.50 ± 3.63	25.69 ± 4.53	44.41^*^	21.64	18.72
EE (kcal/min)	−10%	2.45 ± 0.85	3.29 ± 1.20	2.87 ± 0.68	34.29^*^	17.14	14.63
	−5%	2.51 ± 0.79	3.45 ± 1.34	3.11 ± 0.72	37.45^*^	23.90	10.93
	0%	3.49 ± 0.90	4.46 ± 1.28	4.43 ± 0.88	27.79^*^	26.93	0.68
	5%	5.59 ± 1.15	8.28 ± 1.29	6.26 ± 0.45	48.12^*^	11.99	32.27^*^
	10%	7.86 ± 1.22	11.61 ± 1.20	9.23 ± 1.27	47.71^*^	17.43	25.79^*^
RF (breaths/min)	−10%	27.49 ± 5.74	33.51 ± 6.40	31.62 ± 5.76	21.90^*^	15.02	5.98^*^
	−5%	26.58 ± 4.94	33.38 ± 6.46	32.39 ± 5.98	25.58^*^	21.86	3.06^*^
	0%	27.51 ± 6.54	34.13 ± 5.77	32.29 ± 6.14	24.06	17.38	5.70^*^
	5%	28.51 ± 4.20	37.54 ± 5.18	35.34 ± 4.93	31.67^*^	23.96	6.23
	10%	30.57 ± 3.73	44.67 ± 7.16	40.40 ± 5.61	46.12^*^	32.16	10.57^*^

*NL* No load, *DM* Distributed mode, *CM* Compact mode, *HR* Heart rate, *VO*_*2*_ Oxygen consumption, *EE* Energy expenditure, *RF* Respiratory frequency; ^*^P < 0.05

**Table 2:** Comparison between mode and gradient with their significance, pairwise comparison, *P* value and *F* value for the different parameters

Dependent variable	Independent variable	*F* value	*P* value	Post hoc Bonferroni test (pairwise comparison)
HR (beats/min)	Mode	*F*_(2, 10)_ = 50.92	0.000^*^	M_1_-M_2_^*^ (0.001)
				M_2_-M_3_, NS
	Gradient	*F*_(4, 20)_ = 275.55	0.000^*^	G_1_- G_3_^*^(0.000)
				G_1_- G_4_^*^(0.000)
				G_1_- G_5_^*^(0.000)
				G_2_- G_3_^*^(0.025)
				G_2_- G_4_^*^(0.001)
				G_2_- G_5_^*^(0.000)
				G_3_- G_4_^*^(0.000)
				G_3_- G_5_^*^(0.000)
				G_4_- G_5_^*^(0.001)
	Mode × Gradient	*F*_(8, 40)_ = 12.015	0.000^*^	–
RF (breaths/min)	Mode	*F*_(1.112, 5.560)_ = 17.104	0.000^*^	M_1_-M_2_^*^(0.000)
				M_2_-M_3_, NS
	Gradient	*F*_(4, 20)_ = 19.679	0.001^*^	G_2_- G_5_^*^(0.022)
				G_3_- G_5_^*^(0.027)
				G_4_- G_5_^*^(0.004)
	Mode × Gradient	*F*_(8, 40)_ = 5.137	0.000^*^	–
VO_2_ [ml/(min·kg)]	Mode	*F*_(2, 10)_ = 4.991	0.000^*^	M_1_-M_2_, NS
				M_2_-M_3_, NS
	Gradient	*F*_(4, 20)_ = 963.97	0.031^*^	G_1_- G_3_^*^(0.000)
				G_1_- G_4_^*^(0.000)
				G_1_- G_5_^*^(0.000)
				G_2_- G_3_^*^(0.000)
				G_2_- G_4_^*^(0.000)
				G_2_- G_5_^*^(0.000)
				G_3_- G_4_^*^(0.000)
				G_3_- G_5_^*^(0.000)
				G_4_- G_5_^*^(0.000)
	Mode × Gradient	*F*_(8, 40)_ = 5.663	0.000^*^	–
EE (kcal/min)	Mode	*F*_(2, 10)_ = 4.802	0.000^*^	M_1_-M_2_, NS
				M_2_-M_3_, NS
	Gradient	*F*_(4, 20)_ = 957.28	0.035^*^	G_1_- G_3_^*^(0.000)
				G_1_- G_4_^*^(0.000)
				G_1_- G_5_^*^(0.000)
				G_2_- G_3_^*^(0.000)
				G_2_- G_4_^*^(0.000)
				G_2_- G_5_^*^(0.000)
				G_3_- G_4_^*^(0.000)
				G_3_- G_5_^*^(0.000)
				G_4_- G_5_^*^(0.000)
	Mode × Gradient	*F*_(8, 40)_ = 6.789	0.000^*^	–

*HR* Heart rate, *VO*_*2*_ Oxygen consumption, *EE* Energy expenditure, *RF* Respiratory frequency; ^*^P < 0.05; *NS* Not significant, G_1_. Grade 1, −10%; G_2_. Grade 2, −5%; G_3_. Grade 3, 0%; G_4_. Grade 4, 5%; G_5_. Grade 5, 10%; M_1_. Mode 1, NL; M_2_. Mode 2, distributed mode; M_3_. Mode 3, compact mode; “-”. No data

In terms of HR, the repeated measures ANOVA showed significant difference in Grade (G): *F*_(4, 20)_ = 275.553, and Mode (M): *F*_(2, 10)_ = 50.929. Additionally, a significant interaction was observed for G × M: *F*_(8, 40)_ = 12.015 (*P* < 0.05). Bonferroni’s post hoc test revealed significant interactions between G_1_ and G_3_, G_4_, and G_5_, interactions between G_2_ and G_3_, G_4_, and G_5_; G_3_ with G_4_, and G_5_, as well as interactions between G_4_ and G_5_ and M_1_ with M_2_. The findings of the present study indicate that HR significantly increased from DH gradients to level walking and from level walking to the UH gradients for the DM compared to the NL condition. A similar trend was also followed for the CM. When comparing the DM and CM, the DM exhibited higher responses for all gradients (significant at − 5 and 5% gradients; Table 2).

In terms of VO_2_, the results of the repeated measures ANOVA revealed significant difference in G: *F*_(4, 20)_ = 963.974, M: *F*_(2, 10)_ = 4.991 and in the interaction between G and M: *F*_(8, 40)_ = 5.663. Pairwise comparison via Bonferroni’s post hoc analysis showed significant interactions between G_1_ and G_2_, G_3_, G_4_, and G_5_, interactions between G_2_ and G_3_, G_4_, and G_5_, interactions between G_3_ and G_4_, and G_5_, as well as interactions between G_4_ and G_5_. The data showed that VO_2_ significantly increased from the DH gradients to level walking and from level walking to the UH gradients for the DM compared to the NL condition. A similar pattern was also observed for the CM. The DM had a higher response when compared with the CM; however, the results were insignificant for all gradients (Table 2).

In terms of EE, the results of the repeated measures ANOVA revealed a significant difference in G: *F*_(4, 20)_ = 957.286; and M: *F*_(2, 10)_ = 4.802. The repeated measures ANOVA also showed a significant interaction between G and M: *F*_(8, 40)_ = 6.789 (*P* < 0.05). Bonferroni’s post hoc revealed significant interactions between G_1_ and G_3_, G4, and G5, interactions between G_2_ and G_3_, G_4_, and G_5_, interactions between G3 and G_4_, and G_5_, as well as interactions between G_4_ with G_5_. Similar patterns of changes were observed for EE when the loaded conditions were compared with the NL condition, and the DM had a higher response when compared with the CM, but significant changes were only observed at the 5 and 10% gradients (Table 2).

In terms of RF, the results of the repeated measures ANOVA showed significant difference in G: *F*_(4, 20)_ = 19.679, and M: *F*_(1.112, 5.560)_ = 17.104. The repeated measures ANOVA also showed a significant interaction between G and M: *F*_(8, 40)_ = 5.137 (*P* < 0.05). Bonferroni’s post hoc analysis revealed a significant interaction between G_2_ and G_5_, an interaction between G3 and G_5_, as well as an interaction between G_4_ and G_5_. The interaction between M_1_ and M_2_ was also significantly significant. In the case of RF, the results showed significant increases at the − 10, − 5%, 0, and 10% gradients when the DM was compared with the CM. The DM had a significant increase from the NL condition at the − 10, − 5%, 5, and 10% gradients. However, there were insignificant increases in RF from the DH gradients to level walking, and from level walking to the UH gradients, for the CM compared to the NL condition (Table 2). The authors conducted pairwise comparisons of all conditions, but only the values with significant changes are shown in the table.

## Discussion

This study was conducted to determine if any differences exist in cardio-respiratory responses while carrying the same magnitude load in two different modes over continuously changing DH and UH gradients. The results suggest that HR, VO_2_, EE and RF gradually increased from DH to level and from level to UH gradients when carrying a 31.4 kg load in the DM and CM, compared to the no load condition. Furthermore, the responses were lower in the CM when compared to the DM for all gradients.

According to Soule et al. [15] the distributed mode of load carriage involves higher cardio-respiratory cost in comparison to the compact mode. Moreover, they also found that if the load is well distributed, balanced and placed close to the center of gravity of the body, it costs less energy than placing the load in an unbalanced fashion. Similar observations were reported in the present study. Minetti et al. [20] observed that the energy cost of walking and running increased with increment and decreased with decrement in gradient. This observation and the findings of present study are supported by the outcomes of research conducted by Soule et al. [35]. Furthermore, Malhotra et al. [7] conducted load carriage experiments on school-going children to identify the most cost-effective way of carrying school bags. They concluded that the rucksack was the most cost-effective and efficient mode, and the hand-held carriage was declared as the most inefficient method of carriage in terms of energy expenditure for Indian children. Chatterjee et al. [1] and Pal et al. [9] compared the cardio respiratory parameters of soldiers while carrying loads in CM and DM (10.7 kg and 21.4 kg) on a motor driven treadmill with varying gradients. They concluded that the metabolic cost of the distributed mode was greater than that of the compact mode. In other studies [7, 8], the principle of keeping the load close to the trunk was followed by placing it in a compact mode. In the present study, the compact mode might have utilized the large muscle mass of the back and trunk. Furthermore, the hands remained free in this arrangement, which allowed the body to move in a more balanced manner compared to the distributed mode.

According to Jackson et al. [18] rifle carriage in the hand can be considered as isometric work. Their research showed that when an isometric exercise component was added to a dynamic exercise task, the cardiovascular responses were elevated above levels noted for the dynamic exercise alone. In the existing distributed mode, the rifle is carried in the hand, which disturbs the balance and normal swing of arm during load carriage. Birrel et al. [3] studied the effect of military load carriage on the ground reaction force. They established that rifle carriage restricts the natural swing of the arm, which can modify the vertical and horizontal center of mass of the person. Birrel et al. [36] reported that restricted arm movement in the DM due to rifle carriage in one hand causes increased range of motion of body’s COM. Extra energy expenditure may be required to normalize the COM in this situation. Greater muscular activity of the arm and shoulder carrying rifle may be the key factor behind excess energy cost observed with the distributed mode in the present study. Graves et al. [37] compared a hand held weight to wrist weight and ankle loads and found a 1.36 kg increase in hand or wrist weight increases the energy cost. This observation helps to understand how rifle carriage in the hand can raise the energy expenditure of the participant up to certain extent compared to free hand movements.

Todd et al. [38] found that DH marching with heavy loads showed no reduction in the metabolic demands placed on South African soldiers. Downhill marching also elicited significant decreases in metabolic cost only under lighter load conditions. The physiological response of DH walking in older and younger participants was evaluated by Navalta et al. [39], who found elevated cardiovascular and metabolic responses on the DH gradients in the older individuals compared to their younger counterparts. As hypothesized curvilinear responses for HR, VO_2_, VE and blood pressure were found at the steeper DH gradients. Minimum physiological responses were observed for the − 5% and − 10% grades. The smaller values of the cardio-respiratory variables at − 5% and − 10% gradients that were observed in the present study are corroborated by these findings of Navalta et al. In another study, loads of up to 21.4 kg were carried by Indian soldiers while walking on UH and DH slopes and it was observed that walking at − 10% to − 5% of DH gradients was relatively comfortable in terms of physiological cost. On the other hand, walking on the UH gradients placed a higher demand in terms of VO_2_, HR and EE under both the loaded and unloaded conditions [29]. The CM was found to be more economical in terms of HR, VO_2_, EE and RF responses during heavy load carriage at a walking speed of 3.5 km/h, as compared to the distributed mode for both the DH and UH gradients. In the CM the load was placed closer to the body, which facilitated maintenance of the COM near to its normal position. This might have occurred due to utilization of the major muscle groups of back rather than exploiting the smaller muscle groups (muscles in arm and hand) that may have been used in the distributed mode. The excess demand of cardio-respiratory variables might have resulted in order to compensate for the requirement of smaller muscle groups.

This study is not without limitation; specifically, the data presented here were collected from 8 individuals. A large scale laboratory study, as well as analysis of field conditions with a continuous increase and decrease in slope can advance knowledge in this field and provide more realistic findings.

## Conclusion

Observation from the present study indicate that a higher physiological cost in terms of HR, VO_2,_ EE and RF was associated with distributed mode as compared to the compact mode while walking at 3.5 km/h in all DH and UH gradients (− 10 to 10%). The degree of changes in UH gradients is higher than DH gradients for the distributed mode of load carriage. The knowledge of this study will help in the design and development of new load carriage ensembles, which can reduce the cardio-respiratory burden during heavy load carriage on sloped terrains.

## Abbreviations

BP: Backpack
CM: Compact mode
COM: Center of mass
DH: Downhill
DM: Distributed mode
EE: Energy expenditure
ELCes: Existing load carriage ensembles
G: Grade
HR: Heart rate
HS: Haversack
LCes: Load carriage ensembles
M: Mode
RF: Respiratory frequency
RPE: Rating of perceived exertion
RWL: Relative work load
SL: Sea level
UH: Uphill
VE: Minute ventilation
VO_2_: Oxygen consumption
