# Correction to: Effect of heavy load carriage on cardiorespiratory responses with varying gradients and modes of carriage

**DOI:** 10.1186/s40779-019-0205-x

**Published:** 2019-05-08

**Authors:** Subhojit Chatterjee, Tirthankar Chatterjee, Debojyoti Bhattacharyya, Suranjana Sen, Madhusudan Pal

**Affiliations:** Defence Institute of Physiology and Allied Sciences, Defence Research & Development Organisation, Ministry of Defence, Government of India, Lucknow Road, Timarpur, Delhi, 110054 India

**Correction to: Military Medical Research (2018) 5:26.**

**https://doi.org/10.1186/s40779-018-0171-8**

In the original publication of this article [1], keywords are missing in the article in both online and PDF versions. The keywords for the article are as below:

Keywords: Heavy load carriage, Cardio respiratory responses, Gradients, Soldiers
